# Management of Medication-Related Osteonecrosis of the Jaw (MRONJ) Using Leukocyte- and Platelet-Rich Fibrin (L-PRF) and Photobiomodulation: A Retrospective Study

**DOI:** 10.3390/jcm9113505

**Published:** 2020-10-29

**Authors:** Gianluca Tenore, Angela Zimbalatti, Federica Rocchetti, Francesca Graniero, Domenico Gaglioti, Ahmed Mohsen, Martina Caputo, Marco Lollobrigida, Luca Lamazza, Alberto De Biase, Ersilia Barbato, Umberto Romeo

**Affiliations:** Department of Oral Sciences and Maxillofacial Surgery, Sapienza University of Rome, 00161 Rome, Italy; gianluca.tenore@uniroma1.it (G.T.); angela.zimbalatti@uniroma1.it (A.Z.); graniero.1641033@studenti.uniroma1.it (F.G.); gagliotido@tiscali.it (D.G.); ahmed.mohsen@uniroma1.it (A.M.); martina.caputo@uniroma1.it (M.C.); marco.lollobrigida@uniroma1.it (M.L.); luca.lamazza@uniroma1.it (L.L.); alberto.debiase@uniroma1.it (A.D.B.); ersilia.barbato@uniroma1.it (E.B.); umberto.romeo@uniroma1.it (U.R.)

**Keywords:** autologous platelet concentrates, leukocyte- and platelet-rich fibrin, laser therapy, photobiomodulation, medication-related osteonecrosis of the jaw, oral surgery

## Abstract

Background. The aim of this study was to compare retrospectively the effect of three different treatment protocols on the healing outcome in patients with established medication-related osteonecrosis of the jaw (MRONJ). Methods. A total of 34 MRONJ patients were recruited from the Department database and were divided according to the treatment protocols in a study group (G1) and two control groups (G2 and G3). G1 was treated with antibiotic therapy, surgery, leukocyte- and platelet-rich fibrin (L-PRF), and photobiomodulation; G2 was treated with antibiotic therapy and surgery; G3 was treated with antibiotic therapy and photobiomodulation. Various clinical variables and treatment protocols were analyzed to determine their correlation with the healing outcome at three and six months of follow-up. Results. There was a significant association between the different treatment protocols and the outcomes at both three and six months follow-up (*p* = 0.001 and *p* = 0.002, respectively). No significant association was observed between the outcomes and MRONJ localization, MRONJ stage, duration of drug treatment, gender, diabetes, corticosteroid therapy, smoking habits, underlying disease, and history of chemotherapy at both three and six months follow-up. Conclusions. Our results show that the combination of antibiotic therapy, surgery, L-PRF, and photobiomodulation may effectively contribute to MRONJ management.

## 1. Introduction

Medication-related osteonecrosis of the jaw (MRONJ) is a relatively rare serious adverse drug reaction. It is defined as an area of exposed or probed bone persisting for more than eight weeks in the maxillofacial region in patients with ongoing or a history of treatment with bone-modifying agents and/or angiogenic inhibitor agents [1,2,3]. In 2003, bisphosphonate-related osteonecrosis of the jaw (BRONJ) was reported for the first time in oncologic patients receiving intravenous bisphosphonates [4]. Since then, several cases of osteonecrosis of the jaw were reported being related to bisphosphonates and other medications, such as denosumab, bevacizumab, cabozantinib, and sunitinib [5,6]. Therefore, the American Association of Oral and Maxillofacial Surgeons (AAOMS) has recommended considering this complication being related to medications rather than being related only to bisphosphonates and changed the nomenclature of this complication into MRONJ [3].

The frequency of MRONJ is highly variable and ranges from very rare (less than 1/10,000) to common (1/100 or more), depending on many factors, such as the type of drug, its dose, duration, and the treatment purpose (malignant or osteometabolic diseases) [7].

Although antiresorptive and antiangiogenic therapies improve life expectancy especially of cancer patients, MRONJ may affect the quality of life of the patients due to pain, discomfort, anxiety, depression, impaired speech, swallowing and eating, frequent medical and dental evaluations and treatments, and the possible discontinuation of treatment [8,9]. The management of MRONJ is still controversial with no evidence-based guidelines. The treatment goal is still achieved through control of pain and infection in addition to the minimization of progression or occurrence of bone necrosis [10].

Several therapeutic strategies have been recommended based on the severity of MRONJ, ranging from strictly conservative to aggressive surgical approaches, and the choice between conservative and invasive treatment must be established case by case [11,12,13].

Conservative treatments include maintaining good oral hygiene, periodic dental consultations, antiseptic mouthwashes, and antibiotic therapy. This strategy can stabilize or minimally improve the MRONJ condition [14]. On the other hand, the surgical approach includes the removal of necrotic bone with subsequent normal bone margins. The challenge and limitation of surgical treatment are to determine the precise margins of the osteonecrosis [15].

In the past, surgical treatment was limited to the advanced stages of MRONJ. According to the AAOMS position paper, surgical treatment was not recommended for stages I and II and should be administered to patients with stage III or stage II refractory to nonsurgical treatments [3]. The Italian Society of Maxillofacial Surgery (SICMF) and the Italian Society of Oral Pathology and Medicine (SIPMO) in 2012 recommended conservative surgery in lesions belonging to stages I and II, as defined by both societies, that can offer long-term well-being for patients [16,17]. The complete resolution of the MRONJ lesion is difficult to achieve. Although studies have suggested that surgical treatment is more effective than conservative ones [15,18], some patients still have recurrence or failures that require continuous treatments. Furthermore, randomized controlled studies that investigate whether nonsurgical or surgical treatment provides the best treatment outcome are lacking.

Recent studies have focused on adjuvant therapies such as hyperbaric oxygen, bone morphogenetic proteins, parathyroid hormone, photobiomodulation (PBM), and autologous platelet concentrates (APCs) with the aim to improve healing and reduce recurrence in MRONJ patients [19]. The use of PBM (alone or in combination with other treatments) in MRONJ management has become more widespread in recent years due to the widely reported beneficial effects on tissue healing [20].

PBM improves the reparative process and increases the inorganic matrix of the bone and mitotic osteoblastic index. PBM also increases the motility of human keratinocytes and promotes their increase of collagen type I and vascular endothelial growth factor (VEGF) gene expression [21]. Other PBM positive effects are pain relief, nerve regeneration, and anti-inflammatory action [22]. However, to date, there is no definitive standard protocol for the use of PBM in patients with MRONJ, especially regarding laser parameters (wavelength, delivered energy, energy density, pulse mode, mode of application, power density, and time) [23].

APCs have gained interest in recent years for their regenerative properties in modern dentistry. There are four subtypes of platelet concentrates that are classified by their fibrin and cells properties: pure platelet-rich plasma (P-PRP), leucocyte- and platelet-rich plasma (L-PRP), pure platelet-rich fibrin (P-PRF), and leucocyte- and platelet-rich fibrin (L-PRF). The most commonly used APCs are L-PRP and L-PRF [24]. L-PRF consists of an autologous three-dimensional material with high concentration of leukocytes and growth factors which promote neo-angiogenesis and inflammatory homeostasis, and stimulate collagen production [25]. L-PRF membranes, due to their high density of fibrin fibers, are very resistant to frequent mechanical stresses in the oral cavity and can resist proteolytic degeneration longer [26]. Although the literature reports countless examples of the application of platelet concentrates for preventive and therapeutic purposes in the bone and mucosal tissue defects, their use is recent and evidence-based studies are required to support their application.

The aim of the study was to compare retrospectively the effect of three different treatment protocols on the healing outcomes at three and six months of follow-up in patients with established MRONJ. Additionally, correlation of different clinical characteristics of MRONJ patients with the healing outcome was analyzed.

## 2. Materials and Methods

A single-center retrospective study was conducted on patients enrolled in the MoMax (Oral Medicine and Maxillofacial) project of the Department of Oral Sciences and Maxillofacial Surgery at Policlinico Umberto I, Sapienza University of Rome. All the study procedures were conducted in accordance with the Declaration of Helsinki of 1975, revised in 2013. The study was approved by the Ethics Committee of Sapienza University of Rome (approval number: 981/17).

The following question was structured according to the PICO (patient, intervention, comparison, and outcome) study design:

Which treatment strategy was more effective for the management of MRONJ patients?

The patients were recruited by querying the Department database from January 2019 to March 2020. The inclusion criteria were patients with established MRONJ stage I/II (according to AAOMS staging), with a current or previous history of antiresorptive or antiangiogenic medications, and age ≥18 years old. Patients with MRONJ stage III or IV (according to AAOMS staging), who had undergone any previous treatments of MRONJ, with a history of radiotherapy or metastatic diseases to the maxillofacial region, and/or with generally poor medical conditions were excluded. 

The following data were collected for each patient: Age, gender, underlying disease (malignant or nonmalignant), history of antiresorptive or antiangiogenic medications, site of MRONJ, stage of MRONJ, and type of MRONJ treatments.

A total of 34 patients were recruited and were divided into a study group (G1) and two control groups (G2 and G3). G1 consisted of 13 patients treated with antibiotic therapy, surgery, L-PRF, and PBM. G2 consisted of 8 patients treated with antibiotic therapy and surgery. G3 consisted of 13 patients treated only with antibiotic therapy and PBM.

### 2.1. Treatment Protocols and L-PRF Preparation

Full clinical and radiographic investigations, including computed tomography (CT), were carried out for all the patients to achieve a definite diagnosis of MRONJ. A jaw bone biopsy was performed in case of suspicion of metastasis to confirm the diagnosis [27].

**Figure 1:**
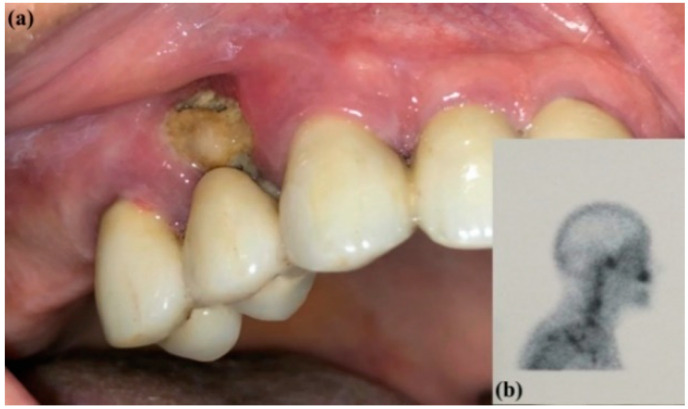
(**a**) Preoperative intraoral clinical view of MRONJ and (**b**) head/neck scintigraphy.

All the treatment protocols are summarized in Table 1. For the patients of G1 and G2, systemic antibiotics (1 g amoxicillin/clavulanic acid and 250 mg metronidazole) were prescribed starting three days before the surgery, two times a day, and ending seven days after. A mouthwash of 0.2% chlorhexidine gluconate was prescribed three times a day starting three days before the surgery and continued for the same period. The patients of G3 received the same antibiotic and antiseptic regimen for seven days in case infection occurred. A preoperative intraoral clinical view of a patient is presented in Figure 1 as a sample of the patients of G1 with a head/neck scintigraphy previously prescribed by the oncologist to monitor the neoplastic disease. The treatment protocol of the same patient will be demonstrated in the subsequent figures.

**Figure 2:**
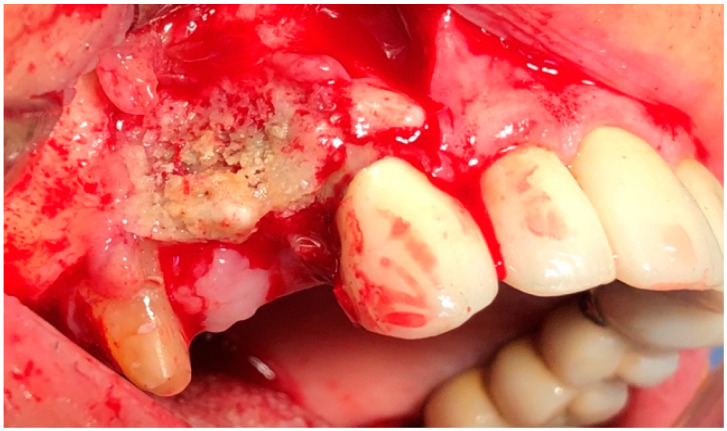
Intraoperative view: incision and elevation of the flap.

**Figure 3:**
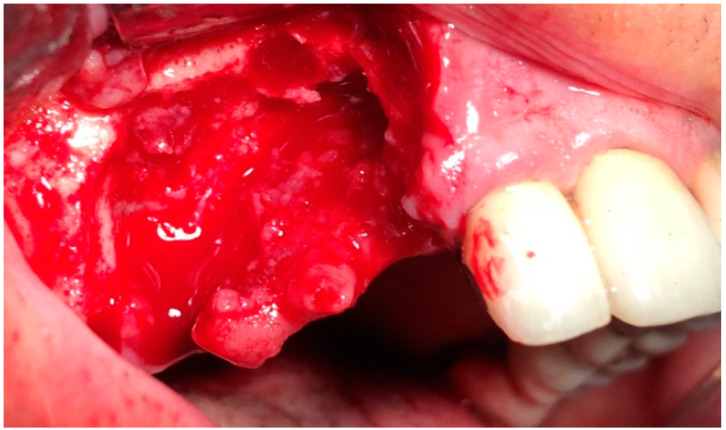
Intraoperative view: resection of necrotic bone.

In G1 and G2, the surgery was performed under local anesthesia using 3% mepivacaine hydrochloride without adrenaline. The surgical protocol consisted of elevation of a full-thickness mucoperiosteal flap to expose the surgical area (Figure 2); sequestrum and granulation tissue were removed using surgical curettes until fresh bleeding was confirmed from the bone (Figure 3). Rotary instruments were used for osteotomy and smoothening out all sharp bone margins.

L-PRF membranes were prepared for the patients of G1. Peripheral blood was collected into 9 mL tubes without anticoagulant and immediately centrifuged at 2700 rpm for 12 minutes (RCFclot = 408 g; RCFmax = 653 g; RCFmin = 326 g) [28] using Instraspin^TM^ centrifuge (Intra-Lock International, Boca Raton, FL, USA) (33° rotor angulation, 50 mm radius at the middle of the tube, 80 mm at the maximum, and 40 mm at the minimum) according to the manufacturer’s instructions.

**Figure 4:**
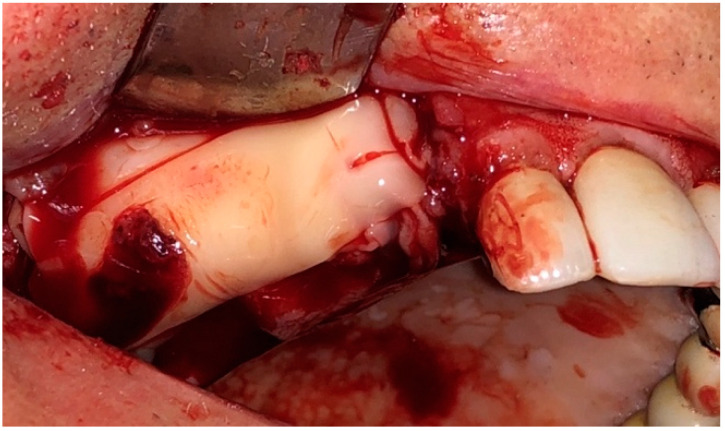
Intraoperative view: Application of L-PRF membranes.

**Figure 5:**
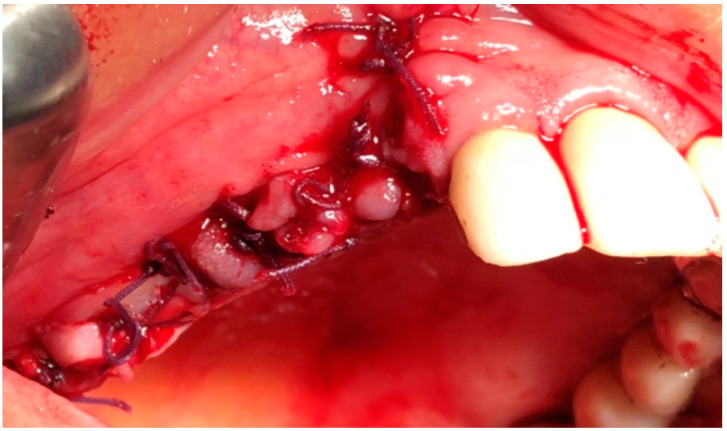
Intraoperative view: sutures.

**Figure 6:**
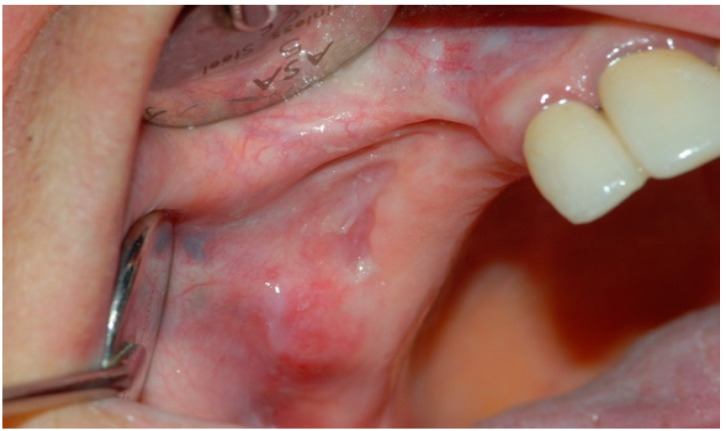
Postoperative intraoral clinical view at 6 months follow-up.

Afterward, L-PRF clots were taken from the tube after discarding the red corpuscles and the acellular plasma at the bottom and top of the tube, respectively; L-PRF membranes were obtained by compressing the clots for five minutes under the weight of a sterile metal plate (Xpression™ Kit, Intra-Lock International, Boca Raton, FL, USA). L-PRF membranes were placed above the bone surface and primary closure of the mucoperiosteal flap was performed (Figure 4 and Figure 5). The sutures were removed 15 days after the surgery in G1 and G2. Five follow-up visits were performed at 7, 15 days, 1, 3, and 6 months for all the patients of the study (Figure 6).

**Figure 7:**
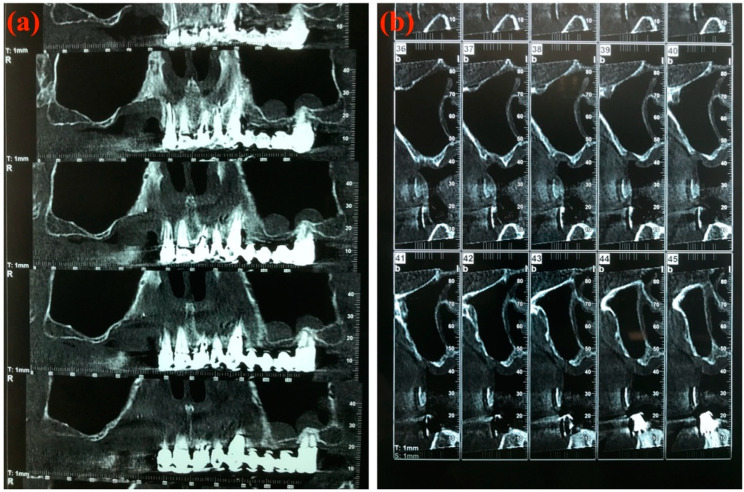
Computed tomography Denta Scan at 6 months of follow-up. (**a**) Panorex view; (**b**) Cross-sectional view.

A CT Denta Scan was performed for all patients at 6 months of follow-up to restage the MRONJ (Figure 7a,b).

### 2.2. PBM Parameters

A multidiodic laser (Lumix C.P.S. Dental, FISIOLINE, Verduno, Cuneo, Italy) emitting simultaneously 650 nm, 810 nm, and 910 nm wavelengths was used for intraoral PBM in G1 and G3. 

The parameters were selected for each group (G1 and G3) according to the manufacturer’s software settings listed in the device. The selected PBM program for G1 was the “Bio-stimulation” program. The PBM program of G3 was the “Necrosis” program.

The patients of G1 were subjected to two sessions per week of PBM for four weeks (total eight sessions). The PBM started five days before the surgical intervention, where two sessions were performed preoperatively. All the lesions were irradiated in scanning and noncontact mode (at ~1 cm distance) with these parameters (per session): total power of 0.6 W, time of 15 min, frequency of 30 kHz, and total energy of 577.4 J. The laser irradiations were performed on the exposed bone and surrounding soft tissues. In G1, preoperatively, the light spot was centered on the margins of MRONJ through the edges of the exposed bone; postoperative, the light spot was centered on the suture line.

In G3, the patients were subjected to a total of eight sessions of PBM (two sessions a week for four weeks) using the same laser device. The lesions were irradiated in the same manner (in scanning and noncontact mode) with these parameters (per session): total power of 1.1 W, time of 15 min, frequency of 80 kHz, and total energy of 531.4 J.

The total laser-irradiated area for both groups (G1 and G3) could not be measured, because the laser was used in a scanning mode and the lesions were of different sizes. All the laser parameters are presented in Table 2.

### 2.3. Assessment of Treatment Outcome

The healing outcome was assessed according to MRONJ response to the treatment as follows: healing when there was an absence of pain and exposed bone, clinical improvement when a transition occurred from a higher to a lower stage, and recurrence when there was a persistence of pain and exposed bone. The data of the three and six months follow-up were obtained for all the patients of the study. The assessment of the healing outcome was obtained through comparing the state of the patients in the programmed follow-up with the initial state of the patients before the intervention.

### 2.4. Statistical Analysis

The R statistical package (version 3.6.1; R Foundation for Statistical Computing, Vienna, Austria) was used for the statistical analysis. Descriptive statistics were obtained for quantitative and qualitative variables. Associations between the treatment outcome at three and six months and some categorial variables such as type of treatment, MRONJ localization, MRONJ stage, duration of drug treatment, gender, diabetes, corticosteroid therapy, smoking habits, underlying disease, and history of chemotherapy were determined using the chi-square test. All values were considered statistically significant when *p* ≤ 0.05.

## 3. Results

The patient’s clinical data are presented in Table 3. The gender distribution of patients was 8 males (23.5%) and 26 females (76.5%), with a mean age of 58.09 years old. The distribution of patients according to the underlying disease was as follows: 15 patients with breast cancer (44.1%), 5 patients with prostate cancer (14.7%), 2 patients with lung cancer (5.9%), 5 patients with multiple myeloma (14.7%), 1 patient with bladder cancer (2.9%), and 6 patients with osteoporosis (17.6%).

Ten patients (29.4%) received denosumab, 5 (14.7%) denosumab and zoledronate, 3 (8.8%) alendronate, 14 (41.2%) zoledronate, one patient (2.9%) received alendronate and zoledronate, and one patient (2.9%) received ibandronate. Time of exposure to antiangiogenic or antiresorptive varied between 8 and 48 months (mean duration of 24.68 months).

Twenty patients (58.8%) were diagnosed with MRONJ in the mandible and 14 patients (41.2%) in the maxilla. Eleven patients (32.4%) were classified as MRONJ stage I and 23 patients (67.6%) were stage II. Seven patients (20.6%) were smokers, 4 patients (11.8%) had diabetes, 28 patients (82.3%) received chemotherapy, and 11 patients (32.4%) received long-term corticosteroid treatments.

There were no significant differences between the groups with regard to gender, age, underlying diseases, type of medications, duration of MRONJ-associated drugs treatment, diabetes, and chemotherapy. Significant differences were found for corticosteroids assumption and smoking habits. Specifically, patients in group 1 were more frequently smokers than those of the other groups, while none of the patients in group 2 were under steroid therapy, differently from groups 1 and 3 (Table 4).

At three months follow-up, complete healing was observed in 27 patients (79.4%), clinical improvement in 5 patients (14.7%), and recurrence in 2 patients (5.9%). At six months follow-up, complete healing was recorded in 22 patients (64.7%), clinical improvement in 7 patients (20.6%), and recurrence in 5 patients (14.7%) (Table 5).

There was a significant association between the healing outcome and the type of treatment protocols at both the three and six months of follow-up (*p* = 0.001 at three months; *p* = 0.002 at six months). All the patients of the study group (G1) showed complete healing for both considered follow-ups.

There was no significant association between the treatment outcome and the following characteristics: MRONJ localization (*p* = 1.000 at three months; *p* = 0.617 at six months), MRONJ stage (*p* = 1.000 at three months; *p* = 1.000 at six months), duration of drug treatment (*p* = 0.737 at three months; *p* = 1.000 at six months), gender (*p* = 0.537 at three months; *p* = 0.515 at six months), diabetes (*p* = 1.000 at three months; *p* = 1.000 at six months), corticosteroid therapy (*p* = 0.523 at three months; *p* = 0.177 at six months), smoking habits (*p* = 0.256 at three months; *p* = 0.334 at six months), underlying disease (*p* = 1.000 at three months; *p* = 1.000 at six months), and history of chemotherapy (*p* = 1.000 at three months; *p* = 1.000 at six months) (Table 6).

## 4. Discussion

MRONJ is a potentially severe complication since it can significantly compromise the patient’s quality of life and reduce the compliance of the patients to antiresorptive or antiangiogenic treatments due to jawbone infections, chronic pain, tooth loss, and compromised function [29,30].

Despite notable progress having been made in understanding and managing this complication, several controversial aspects remain, regarding pathogenesis, diagnosis, and treatment of MRONJ. Therefore, prevention becomes fundamental for the management of MRONJ and should involve the cooperation between different health providers such as physicians, drug prescribers, dentists, and oral hygienists.

Primary prevention prior to commencing treatment with MRONJ, including dental screening, and treatments of oral diseases, aims to eliminate oral and dental risk factors, maintaining good oral health and reducing the risk of the onset of pathological conditions or any other negative event [31].

To date, agreed guidelines for medical and/or surgical treatment for MRONJ have not been officially drawn. A multidisciplinary approach with a detailed assessment of the patient’s general conditions and life expectancy is indispensable. The literature reports several treatments, isolated or combined, including antibiotics, local irrigation with antimicrobial agents, debridement, and sequestrectomy. Surely, the choice of treatment should be considered on a case-by-case basis.

Even though conservative nonsurgical treatment may be able to slow down MRONJ progression and alleviate superinfection of the exposed bone [32], surgical treatment protocols have returned superior results, in particular regarding the complete mucosal healing [33,34].

A recent literature review by AlDhalaan et al. reported that conservative nonsurgical treatment should be restricted to patients who cannot undergo surgical treatment, for example, for pathological conditions, with high operative risk of neoplastic diseases considerably undermining the life expectancy [10].

In the past, many consensus reports have recommended that any type of surgery should be deferred as long as possible and any surgery to cure MRONJ should be performed only in symptomatic patients or for extensive disease [35,36]. By contrast, according to the recent recommendations by SIPMO/SICMF (2020), it is suggested to anticipate surgical treatment, whenever indicated, to reduce the surgical burden for MRONJ patients and increase the likelihood of long-term healing [30].

A minimally surgical approach with laser surgery has been described as one of the most promising laser systems for bone surgery [37]. Several studies have shown positive results related to wound healing in patients with MRONJ [38,39,40]. Stubinger et al. reported that 100% of patients treated with the Er:YAG laser obtained complete healing of the lesions [41].

According to recent studies, both conservative medical and surgical approaches might be optimized by adding an adjuvant treatment such as PMB, platelets concentrates, hyperbaric oxygen, ozone therapy, or lactoferrin, to improve the healing process [18,19,42,43].

Literature on ozone therapy, teriparatide, and lactoferrin is scarce, with a lack of controlled studies. According to Calvani et al., the difference in the healing time when using lactoferrin versus conservative medical therapy was a few weeks and up to three months, respectively [44].

No protocols are established regarding the number and frequency of PMB and the use of platelets concentrates.

The present study analyzed retrospectively the effect of three different protocols for MRONJ management, including a combined protocol consisting of drug therapy, surgical therapy, L-PRF, and PBM. 

Many authors suggested the use of PBM application due to its beneficial effects on tissue healing [45]. According to Garavello-Freitas et al., a two-step mechanism is involved in the interaction of PBM and the bone repair process; the first is probably related to the activation of osteoblasts to produce bone matrix. In a subsequent stage, an inhibitory photobiological mechanism would decrease the activity of the osteoblasts and PBM would stimulate osteoclast activity to promote bone resorption and remodeling [46]. Also, Yamamoto et al. affirm that PBM plays a role in stimulating the proliferation of osteoblasts through the enhancement of the minichromosome maintenance complex (MCM) family gene expression [47].

Regarding the PBM effect in soft tissue healing, a study conducted by Lee et al. on a culture of alendronate-treated oral keratinocytes demonstrated that PBM inhibited apoptosis caused by alendronate. Moreover, after alendronate treatment, PBM promoted cell viability, cell migration, protein production associated with angiogenesis, and wound healing [48]. Tenore et al. reported a significant reduction of pain (more than 2 NRS values) in 25 MRONJ patients treated with a PBM cycle consisting of five sessions [49].

The properties of APCs appear particularly useful in MRONJ surgical therapy, where the lack of vascularization represents one of the major factors on the pathogenesis of MRONJ. In addition, APCs are biocompatible, simple, malleable, and safe products suitable for use in oral surgery [50]. Mauceri et al. conducted a study on 10 BRONJ patients treated with laser-assisted surgery together with PRP. The authors observed with the combined approach a successful outcome of 80% (30% of patients with no clinical and radiological signs of BRONJ relapse and 50% with clinical improvement) [51].

Unlike PRP, L-PRF persists in the site of application, providing superior prolonged action (over 7 to 28 days), compared with other preparations [52]. Furthermore, Inghingolo et al. concluded that L-PRF acts as a barrier membrane between the alveolar bone and the oral cavity [53].

Kim et al. reported the application of L-PRF in the treatment of 34 BRONJ patients, with a complete resolution in 26 (76%) cases, a delayed resolution in 6 (18%) cases, and no resolution in 2 (6%) cases [54]. Maluf et al. reported complete healing in two cases of MRONJ stage II treated with bone debridement combined with the application of L-PRF [55]. Although most of the studies reported that APCs might be beneficial for reducing the postsurgical occurrence and recurrence of MRONJ, a review conducted by Del Fabbro et al. reported substantial lack of evidence for the use of APCs in postextraction sockets due to short follow-up period and heterogeneity of the studies considered [50].

Recently, studies have been conducted on the use of bone morphogenetic protein-2 (BMP-2) for MRONJ treatment. BMP-2 plays a crucial role in the development of bone and cartilage and induces osteoblast differentiation. Considering that the pathogenesis of MRONJ involves oversuppression of bone remodeling, BMPs-2 are expected to promote the healing of bone in MRONJ patients by enhancing bone remodeling [56]. Park et al. proposed the hypothesis that the simultaneous application of L-PRF and BMP-2 stimulates soft tissue healing and bone remodeling, thus contributing to success in MRONJ management. In their study on 55 MRONJ patients (25 treated with a single application of L-PRF and 30 treated with simultaneous application of L-PRF and rh recombinant human BMP-2), MRONJ healing was significantly improved in the PRF-plus-BMP group compared with the PRF group (odds ratio = 4.172; 95% confidence interval, 1.165 to 14.944; *p* = 0.028), indicating that the additional use of BMP-2 significantly improved MRONJ healing. Furthermore, the application of BMPs-2 leads to the early resolution of MRONJ [57].

In our study, most of the patients that showed complete healing were treated with antibiotic therapy, surgery, L-PRF, and PBM. The association of microbial growth control through using systemic antibiotics, the positive effect of L-PRF on tissue healing, and the biophysical properties of PBM proved to be successful in the management of MRONJ. Our results are similar to those of Martins et al. that retrospectively compared the impact of the PBM and PRP protocol with a pharmacological and surgical therapy on wound healing in 22 BRONJ patients. They obtained higher rates of success, in terms of mucosal wound healing, in patients surgically treated with the PBM and PRP protocol [58].

It is difficult to identify standardized PBM parameters for a treatment modality. This is because of a lack of knowledge of the exact effect of each parameter and lack of uniform reports of the physical and biological variables such as type of laser, output power, frequency, time of application, and the histological differences between treated tissues [22,59,60]. Therefore, it was decided to select the used two programs with these parameters for G1 and G3 groups from the manufacturer’s setting list of this device in order to be a step forward in our future studies for the necessity of standardization.

There were some limitations that were observed in our study and should be considered, including the limited sample size, the retrospective nature of the study, the inclusion of only MRONJ patients with stage I and stage II, and the presence of confounding factors, such as applied comedications (e.g., corticosteroids and/or different chemotherapy) in some patients.

In addition, the PBM parameters were not identical in the two groups (G1 and G3). The two different parameters of PBM were because of two reasons. First, the purpose of PBM application was different between G1 and G3 groups. In G1, the treatment protocol was the surgical intervention and the PBM was applied as an adjuvant treatment to the surgical approach. Thus, the program “Bio-stimulation” was chosen to promote biostimulating properties, analgesia, and wound healing, and to optimize the clinical evolution and treatment time, while the PBM in G3 was applied as an adjuvant treatment to the symptomatic therapy (oral antibiotics, antiseptic mouth rinses, pain relief, and infection control) as the exposed necrotic bone was not surgically removed for these patients (of G3), thus the program “Necrosis” in this situation would have been more appropriate and was chosen. Second, the study was a retrospective study and the patients were recruited in these two groups retrospectively, and the patients were not employed for testing with and without surgical intervention, where the first therapeutic approach was chosen on the basis of the most updated guidelines available at the time of treatment and according to the patient’s systemic conditions and consensus. These issues should be also acknowledged as a limitation of the study and considered in the interpretation of the results.

## 5. Conclusions

Despite the growing awareness of MRONJ, a standardized treatment protocol is still missing. Our results showed that significantly better results were obtained when the surgical treatment was combined with L-PRF and PBM. Further research with a larger sample size including all the MRONJ stages is necessary to confirm our promising results.

## Figures and Tables

**Table 1:** Treatment protocols of the study group and the control groups.

	Treatment Protocols		
	Preoperative	Intraoperative	Postoperative
**Study Group (G1)**	Intraoral PBM started five days before the surgical intervention, where two sessions were performed preoperatively. Antibiotic and antiseptic regimen starting three days before surgery: - 1 g amoxicillin/clavulanic acid and 250 mg metronidazole orally two times a day. Mouthwash with 0.2% chlorhexidine gluconate three times a day.	Sequestrectomy of necrotic bone or superficial debridement/curettage or corticotomy/surgical removal of alveolar and/or cortical bone. Preparation and positioning of L-PRF membranes.	Same antibiotic and antiseptic regimen continued for seven days after surgery. Intraoral PBM continued two times a week (total of eight PBM sessions).
**Control Group (G2)**	Antibiotic and antiseptic regimen starting three days before surgery: - 1 g amoxicillin/clavulanic acid and 250 mg metronidazole orally two times a day. Mouthwash with 0.2% chlorhexidine gluconate three times a day.	Sequestrectomy of necrotic bone or superficial debridement/curettage or corticotomy/surgical removal of alveolar and/or cortical bone.	Same antibiotic and antiseptic regimen continued for seven days after surgery.
**Control Group (G3)**	Started the same antibiotic and antiseptic regimen of G1 and G2 in case the infection occurred.	Eight sessions of intraoral PBM (two sessions a week for four weeks).	Same antibiotic and antiseptic regimen in case the infection occurred.

**Table 2:** Photobiomodulation parameters in study group (G1) and control group (G3).

Laser PBM Settings	Study Group (G1)	Control Group (G3)
**Manufacturer**	FISIOLINE	
**Model identifier**	Lumix^®^ C.P.S. ^®^ Dental (Multidiodic laser)	
**Number and type of emitters**	Three wavelengths, visible and infrared GaAs	
**Wavelengths**	650 nm, 810 nm, and 910 nm	
**Pulse mode**	For visible 650 nm: continuous mode, for 810 nm: continuous modulating, and for 910 nm: 30 kHz	For visible 650 nm: continuous mode, for 810 nm: continuous modulating, and for 910 nm: 80 kHz
**Spot size**	~0.5 cm^2^	
**Exposure duration**	15 minutes	
**Application technique**	Scanning in a defocused mode	
**Total irradiation energy per session**	577.4 J	531.4 J
**Number and frequency of treatment sessions**	Two sessions per week starting five days before the surgery and continued for four weeks (total of eight sessions)	Two sessions per week and continued for four weeks (total of eight sessions)

**Table 3:** Clinical data of the patients included in the study.

Patients	Age	Gender	Underlying Disease	Antiresorptive or Antiangiogenic	Admin. Method	Duration of Drug (Months)	Cort.	CT	Diabetes	Smoking	MRONJ Stage *	MRONJ Localization
**Study Group (G1)**												
1	73	M	Lung cancer	Denosumab	I.M	29	No	Yes	No	Yes	II	Mand
2	74	M	Bladder cancer	Zoledronate	I.V	21	Yes	Yes	No	Yes	II	Mand
3	78	M	Prostate cancer	Zoledronate	I.V	39	Yes	Yes	Yes	Yes	II	Max
4	77	M	Prostate cancer	Denosumab	I.M	36	No	Yes	No	Yes	II	Mand
5	74	F	Multiple myeloma	Zoledronate	I.V	22	No	Yes	No	No	II	Max
6	58	F	Breast cancer	Denosumab	I.M	12	Yes	Yes	No	No	II	Mand
7	82	M	Prostate cancer	Zoledronate	I.V	30	No	Yes	No	No	II	Mand
8	68	F	Breast cancer	Denosumab	I.M	72	No	Yes	No	No	I	Mand
9	82	F	Multiple myeloma	Zoledronate-denosumab	I.V/I.M	97	No	Yes	No	No	II	Max
10	58	F	Osteoporosis	Alendronate	Oral	6	No	No	No	Yes	I	Mand
11	67	F	Osteoporosis	Zoledronate-alendronate	I.V/Oral	60	No	No	No	Yes	I	Mand
12	69	F	Osteoporosis	Alendronate	Oral	60	No	No	No	No	II	Max
13	79	F	Multiple myeloma	Zoledronate	I.V	10	Yes	Yes	No	No	II	Mand
**Control Group (G2)**												
14	45	F	Breast cancer	Zoledronate	I.V	24	No	Yes	No	No	II	Mand
15	84	F	Osteoporosis	Ibandronate	Oral	96	No	No	Yes	No	II	Mand
16	51	F	Breast cancer	Zoledronate- denosumab	I.V/I.M	31	No	Yes	No	No	II	Mand
17	56	F	Breast cancer	Zoledronate- denosumab	I.V	62	No	Yes	No	No	II	Max
18	61	F	Breast cancer	Zoledronate- denosumab	I.V	30	No	Yes	No	No	II	Max
19	60	F	Breast cancer	Denosumab	I.V	36	No	Yes	No	No	I	Max
20	65	F	Breast cancer	Denosumab	I.M	23	No	Yes	No	No	II	Max
21	74	F	Breast cancer	Zoledronate	I.V	24	No	Yes	No	No	I	Mand
**Control Group (G3)**												
22	72	M	Prostate cancer	Zoledronate	I.V	33	Yes	Yes	No	No	I	Max
23	74	F	Multiple myeloma	Zoledronate	I.V	30	Yes	Yes	No	No	II	Max
24	59	F	Breast cancer	Zoledronate	I.V	24	Yes	Yes	No	No	II	Max
25	81	M	Multiple myeloma	Zoledronate	I.V	6	Yes	Yes	Yes	No	II	Mand
26	84	F	Osteoporosis	Alendronate	Oral	84	No	No	No	No	II	Max
27	92	M	Prostate cancer	Zoledronate-denosumab	I.V/I.M	48	No	Yes	No	No	II	Max
28	65	F	Lung cancer	Zoledronate	I.V	21	No	Yes	No	Yes	II	Max
29	61	F	Breast cancer	Zoledronate	I.V	24	No	Yes	No	No	I	Mand
30	81	F	Osteoporosis	Denosumab	I.M	48	Yes	No	No	No	I	Mand
31	62	F	Breast cancer	Denosumab	I.M	24	Yes	Yes	Yes	No	I	Mand
32	55	F	Breast cancer	Zoledronate	I.V	24	Yes	Yes	No	No	II	Max
33	78	F	Breast cancer	Denosumab	I.M	15	No	Yes	No	No	I	Mand
34	71	F	Breast cancer	Denosumab	I.M	24	No	Yes	No	No	I	Mand

* MRONJ stage according to AAOMS classification; M: Male; F: Female; Admin.: Administration; I.V: Intravenous; I.M: Intramuscular; Cort.: Corticosteroids use; CT: Chemotherapy; Mand: Mandible; Max: Maxilla.

**Table 4:** Baseline characteristics of the patients included in the study.

Variables	Groups			Total	*p*
	G1	G2	G3		
**Median Age (IQR)**	74 (67.5–78.5)	60.5 (52.25–71.75)	72 (61.5–81)	71.5 (60.75–78.25)	0.136 *
**Gender**					
**Female**	8	8	10	26	0.13
	61.50%	100.00%	76.90%	76.50%	
**Male**	5	0	3	8	
	38.50%	0.00%	23.10%	23.50%	
**Neoplastic diseases**	10	7	11	28	0.796
	76.90%	87.50%	84.60%	82.40%	
**Osteoporosis**	3	1	2	6	0.796
	23.10%	12.50%	15.40%	17.60%	
**Antiresorptive and/or antiangiogenic medications**					
**Bisphosphonate**	7	3	8	18	0.53
	53.80%	37.50%	61.50%	52.90%	
**both**	2	3	1	6	
	15.40%	37.50%	7.70%	17.60%	
**Denosumab**	4	2	4	10	
	30.80%	25.00%	30.80%	29.40%	
**Median MRONJ-related drugs therapy duration (months—IQR)**	30 (16.5–60)	30.5 (24–55.5)	24 (22.5–40.5)	29.5 (22.75–48)	0.599 *
**Corticosteroids**	4	0	7	11	0.037
	30.80%	0.00%	53.80%	32.40%	
**Chemotherapy**	10	7	11	28	0.796
	76.90%	87.50%	84.60%	82.40%	
**Diabetes**	1	1	2	4	0.829
	7.70%	12.50%	15.40%	11,80%	
**Smoking habit**	6	0	1	7	0.014
	46.20%	0.00%	7.70%	20.60%	
**MRONJ Stage**					
**1**	3	2	6	11	0.399
	23.10%	25.00%	46.20%	32.40%	
**2**	10	6	7	23	
	76.90%	75.00%	53.80%	67.60%	
**MRONJ localization**					
**mandible**	9	4	6	19	0.461
	69.20%	50.00%	46.20%	55.90%	
**maxilla**	4	4	7	15	
	30,80%	50.00%	53.80%	44.10%	
**Total**	**13**	**8**	**13**	**34**	
	**100.00%**	**100.00%**	**100.00%**	**100.00%**	

IQR: interquartile range; * Kruskal–Wallis test.

**Table 5:** Outcome of the three different groups at three and six months of follow-up.

Groups	Treatment Outcome					
	At 3 Months Follow-Up *n* (%)			At 6 Months Follow-Up *n* (%)		
	Healing	Improvement	Recurrence	Healing	Improvement	Recurrence
**Study Group (G1)**	13 (100)	0	0	13 (100)	0	0
**Control Group (G2)**	8 (100)	0	0	4 (50)	1 (12.5)	3 (37.5)
**Control Group (G3)**	6 (46.2)	5 (38.5)	2 (15.4)	5 (38.5)	6 (46.2)	2 (15.4)

**Table 6:** MRONJ outcome in relation with type of MRONJ treatment, MRONJ localization, MRONJ stage, duration of drug treatment, gender, diabetes, corticosteroid therapy, smoking habit, underlying disease, and history of chemotherapy.

Variables	Treatment Outcome					
	At 3 Months Follow-Up			At 6 Months Follow-Up		
	Df	X^2^	*p*	Df	X^2^	*p*
**Type of MRONJ treatment**	4	14.239	0.001	4	15.954	0.002
**MRONJ localization**	2	0.069	1	2	1.1	0.617
**MRONJ stage**	2	0.51	1	2	0.182	1
**Duration of drug treatment**	2	1.086	0.737	2	0.277	1
**Gender**	2	1.584	0.537	2	1.809	0.515
**Diabetes**	2	0.604	1	2	0.521	1
**Corticosteroid therapy**	2	1.576	0.523	2	3.173	0.177
**Smoking habit**	2	2.399	0.256	2	2.417	0.334
**Underlying disease**	2	0.742	1	2	0.078	1
**History of chemotherapy**	2	0.742	1	2	0.078	1

Df: Degrees of freedom; X2: Values determined using the chi-square test.
